# Early Stages of Aluminum-Doped Zinc Oxide Growth on Silicon Nanowires

**DOI:** 10.3390/nano12050772

**Published:** 2022-02-25

**Authors:** Giovanni Borgh, Corrado Bongiorno, Salvatore Cosentino, Antonino La Magna, Salvatore Patanè, Silvia Scalese, Antonio Terrasi, Giacomo Torrisi, Rosaria A. Puglisi

**Affiliations:** 1 Department of Mathematics and Computer Science, Physics and Earth Science (MIFT), University of Messina, Viale F. Stagno d’Alcontres, 31, 98166 Messina, Italy; giovanni.borgh@unime.it (G.B.); salvatore.patane@unime.it (S.P.); 2 Institute for the Microelectronics and Microsystems (IMM), National Research Council (CNR), Catania Headquarters (HQ), Strada Ottava 5, Zona Industriale, 95121 Catania, Italy; corrado.bongiorno@imm.cnr.it (C.B.); antonino.lamagna@imm.cnr.it (A.L.M.); silvia.scalese@imm.cnr.it (S.S.); 3 Institute for the Microelectronics and Microsystems (IMM), National Research Council (CNR), Catania University, Via S. Sofia 64, 95123 Catania, Italy; salvo.cosentino@gmail.com (S.C.); antonio.terrasi@ct.infn.it (A.T.); giacomo.torrisi@gmail.com (G.T.); 4 Department of Physics and Astronomy, University of Catania, Via S. Sofia 64, 95123 Catania, Italy

**Keywords:** AZO, silicon, nanowires, STEM, EELS

## Abstract

Aluminum-doped zinc oxide (AZO) is an electrically conductive and optically transparent material with many applications in optoelectronics and photovoltaics as well as in the new field of plasmonic metamaterials. Most of its applications contemplate the use of complex and nanosized materials as substrates onto which the AZO forms the coating layer. Its morphological characteristics, especially the conformality and crystallographic structure, are crucial because they affect its opto-electrical response. Nevertheless, it was difficult to find literature data on AZO layers deposited on non-planar structures. We studied the AZO growth on silicon-nanowires (SiNWs) to understand its morphological evolution when it is formed on quasi one-dimensional nanostructures. We deposited by sputtering different AZO thicknesses, leading from nanoclusters until complete incorporation of the SiNWs array was achieved. At the early stages, AZO formed crystalline nano-islands. These small clusters unexpectedly contained detectable Al, even in these preliminary phases, and showed a wurtzite crystallographic structure. At higher thickness, they coalesced by forming a conformal polycrystalline shell over the nanostructured substrate. As the deposition time increased, the AZO conformal deposition led to a polycrystalline matrix growing between the SiNWs, until the complete array incorporation and planarization. After the early stages, an interesting phenomenon took place leading to the formation of hook-curved SiNWs covered by AZO. These nanostructures are potentially very promising for optical, electro-optical and plasmonic applications.

## 1. Introduction

Silicon-nanowires (SiNWs) are interesting nanostructures whose properties are useful for many applications in nano-electronics. They are used to engineer the emitter of a new photodiodes class with a non-planar configuration [1], can be exploited as architectures to make radial and axial junctions [2,3,4], can act as light-trapping structures to improve the optical path [5], and can be used as powerful building blocks to realize high mobility channels in field effect transistors [6], biosensors [7] and catalysts [8]. A transparent electrode is required in many of these technologies; the transparent conductive oxides (TCOs) are the most often used materials [9,10]. TCOs must have a bandgap greater than 3.1 eV to be effective in transmitting photons of visible light without exciting electrons from the valence to the conduction band, granting improved structural, thermal and optical properties [11]. TCOs integration in the final devices is difficult and currently represents a challenge, as it must meet some conditions such as being conformal to the nanostructure geometry, as well as to ensure at the same time good opto-electrical and mechanical properties. Moreover, it should be made of non-toxic, eco-friendly, and inexpensive materials. Among these, aluminum-doped zinc oxide (AZO) has been gaining ground for large-scale applications. Solar cells, flat panel displays, and light-emitting diodes are just some examples of where AZO is used [12,13,14]. Its success is mainly due to its lateral conductivity improvement as well as its good optical and surface passivation properties [15]. Moreover, it has a direct wide bandgap of 3.37 eV and a large exciton binding energy of ~60 meV [16]. It is also mechanically resistant [17], exhibits an intrinsic n-type behavior due to crystal defects and allows heavy doping [18], in addition to being abundant, inexpensive and non-toxic [19]. The TCOs morphology affects the transparency of the component and the excitonic propagation length. Indeed, crystallinity is needed for both transparency and efficient exitonic propagation, which controls the electrical conductivity [20]. For large-scale nano electro-optical applications based on SiNWs, efficient passivation and chemical stability of the active area are essential, as are good electrical characteristics. Therefore, it is useful to understand the interaction between AZO and the structured substrate and how it evolved SiNWs from the beginning of the deposition, because this determines the final characteristics of the material. Currently, there is no literature available on the early deposition phases, especially when substrates with complex shapes, as in the case of SiNWs, are involved.

In this work, we synthesized and studied a system composed of the combination of AZO and SiNWs; we focused on the early stages of AZO formation on the nanostructures. SiNWs arrays were grown via inductively coupled chemical vapor deposition (CVD) through the vapor liquid solid (VLS) mechanism, using Au clusters as catalysts [21]. The coating of SiNWs with AZO was pursued by sputtering. Sputtering is known to allow for faster processing and lower costs than other techniques and does not require ultra-high vacuum conditions; indeed, it is the most used technique for TCO fabrication. We aimed at investigating the nucleation and growth mechanisms and how the formation of the layer evolves as the amount of deposit increases. We showed results on AZO-coated SiNWs in terms of chemical composition of the deposited material and its morphology, evaluating its conformality with respect to the substrates. The oxide structure was analyzed at the atomic level, observing its crystalline nature and defining its phase from the first deposition steps up to a hundred nanometers. In the early stages, the deposited AZO assembled on the nanowires in the form of nanoclusters decorating them along their entire length. This study provides important insights for the application of AZO-decorated SiNWs as transparent electrodes or innovative plasmon metamaterials. The plasmonic behavior of SiNWs in the near UV and visible regions is not yet completely clear, despite being extremely interesting regions to many fields of applications. It is known that the plasmonic characteristics and, therefore, the functionality of devices that exploit this phenomenon are sensitive to the material that surrounds the structure through its refractive index. Hence, we expected that it could be possible to modulate the plasmonic resonances of these nano systems by controlling the morphology and the amount of deposited AZO. The above properties together with the low costs open the way, in the future, for large-scale use of such SiNWs/AZO combined systems for plasmonic functionalities and photovoltaics.

## 2. Materials and Methods

The substrate used was CZ <100> 13 Ωcm p type 6″ Si wafer. The samples were cleaned in a sonicating bath of acetone, ethanol and water for about 5 min each. Then, a dip in HF (1%) was performed to remove the native oxide film. Once dried, the substrate was loaded into the sputter chamber for the Au deposition at a pressure of 5 × 10^−5^ mbar and current of 10 mA for 60 s. The equivalent Au thickness deposited was about 2 nm (monitored by in situ quartz balance). After a second brief HF dip, the substrate was transferred into the CVD chamber, where it was heated at 380 °C for 1 h under vacuum to allow for the formation of the euthectic. Then, the monosilane (SiH_4_) and Ar were introduced in the CVD chamber and the SiNWs growth took place at 380 °C for 30 min with a pressure equal to 20 mTorr and a plasma power of 20 W. The SiH_4_/Ar ratio was fixed to 30. Once the SiNWs growth was complete and the sample was exposed to the atmosphere, a compliant layer of native silica of about 2 nm thick surrounding the wires was immediately produced. After VLS growth, SiNWs underwent a two-step procedure for the Au removal: 5 min HF etch, then, a gold etch containing sodium iodide (NaI) and iodine (I_2_) for 4 min. After, the wafer was cut into 2 cm^2^ square pieces used for the AZO deposition. Each sample was introduced individually into the sputter deposition chamber. In the current work, a commercial AZO disk as target for the sputtering was used, with zinc oxide–aluminum oxide, 97-3 wt%, 50.8 mm dia. × 3.18 mm thick, 99.99% purity, 97% Zn oxide, 3% Al oxide, % by weight. No rotation was imposed to the substrates during the depositions; their surface was parallel with respect to the source. The wafer holder was not purposely heated. The plasma was triggered by radio frequency (RF). The pressure in the chamber was equal to about 3 × 10^−3^ mbar; the power was equal to 50 W. The thickness of the deposited material was chosen based on a previous calibration that returned a deposition rate equal to 0.04 nm/s. The process times chosen varied from a few seconds to about 4 h for the thicker layers. Four different AZO equivalent thicknesses were deposited: 2 nm, 20 nm, 100 nm and 500 nm. The samples obtained were characterized by scanning electron microscopy (SEM), and transmission electronic microscopy (TEM). The SEM analyses were obtained by a Supra35 FE-SEM (Zeiss, Oberkochen, Germany). The TEM analyses were performed by using a JEOL ARM200F (JEOL Ltd., Tokyo, Japan) Cs-corrected microscope, equipped with a cold-field emission gun and operating at 200 keV. Micrographs were acquired by high-angle annular dark field (HAADF). The diffraction analysis was used to study the crystal lattice of the deposit. Energy dispersive X-ray (EDX) analysis and electron energy loss spectroscopy (EELS) provided information on the chemical composition of the clusters and of the layers. The EELS measurements were obtained by using a GIF Quantum ER system (Gatan AMETEK, Pleasanton, CA, USA). EELS spectra were acquired with the EELS tool through Gatan DigitalMicrograph software Version 3.4 (Gatan AMETEK, Pleasanton, CA, USA).

## 3. Results and Discussion

**Figure 1:**
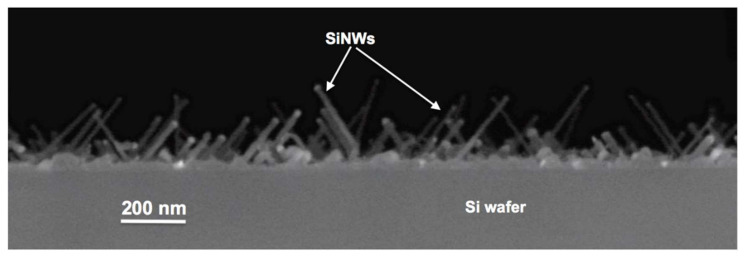
SEM micrograph in cross view of the as grown SiNWs array after the CVD deposition used as substrate for the AZO depositions.

Figure 1 shows the SiNWs array obtained after the CVD growth and used as a substrate for the AZO depositions. SiNWs had diameters ranging between 5 and 35 nm and lengths between 50 and 500 nm, in some cases reaching the micron. Unlike other growth methods [22], the SiNWs grown with our method were straight and oblique due to the crystallographic plane orientation effect of the Si substrate on which they were grown [23,24].

**Figure 2:**
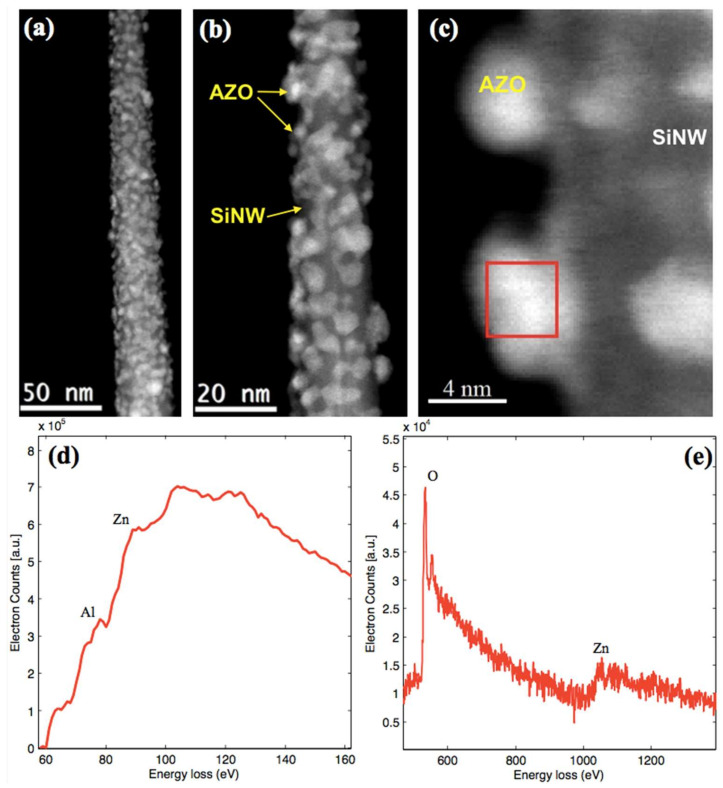
(**a**–**c**) STEM images at different magnifications for the SiNW/AZO system. The equivalent thickness of the deposited AZO layer is 2 nm; the diameter of the Si-NW imaged is about 20 nm. The AZO clusters diameter is smaller than 10 nm. (**d**,**e**) EELS spectra extracted from significant region as shown by the red rectangle in (**c**).

Figure 2a–c show one Si-NW imaged in HAADF STEM mode from the sample subjected to the AZO deposition of 2 nm equivalent thickness, observed at different magnifications. Figure 2a reveals a clear deposit made of quasi 0-dimensional nanoclusters with sizes smaller than 10 nm and size-distributed on the surface.

Figure 2d,e report the EELS spectra in the most significant energy ranges, i.e., from 70 to 160 eV and from 500 to 1400 eV, respectively, for the same Si-NW, extracted from the cluster region as indicated by the red box in the dark field STEM image in Figure 2c. The signal in Figure 2d at energies larger than 78 eV is due to Al *L*-edge, while the peak at about 90 eV is relative to the Zn3p [25]. The doublet located at 537 and 556 eV in Figure 2e is due to the O1s and the signals at about 1022 and 1045 eV are due to Zn2p 3/2 and ½, respectively [26]. The EELS spectrum backgrounds were subtracted using a power law model.

The data presented in the literature on the O peak, for planar AZO films, were mainly obtained by XPS analysis with different energy scale calibration [27]. So, our results obtained by EELS spectroscopy provide a new useful reference. The presence of the Zn and O EELS peaks indicated that the grain was composed of ZnO; the Al signal observed in the cluster structure confirmed the presence of the metal atoms inside the grain, suggesting that the sputtered material was, in fact, AZO and not simple ZnO, even at this low deposition thickness. This result was surprising, as the small size of the AZO clusters and the small amount of dopant made their characterization difficult; it is also statistically possible to find undoped nanostructures [28,29,30,31,32,33]. Based on findings in the literature, the Al signal at 78 eV in Figure 2d can be ascribed to the Al-O bond, as expected for the Al-doped ZnO, which presents an atomic structure where the Al^3+^ ions substitute Zn^2+^ ions in the lattice [26,34,35].

**Figure 3:**
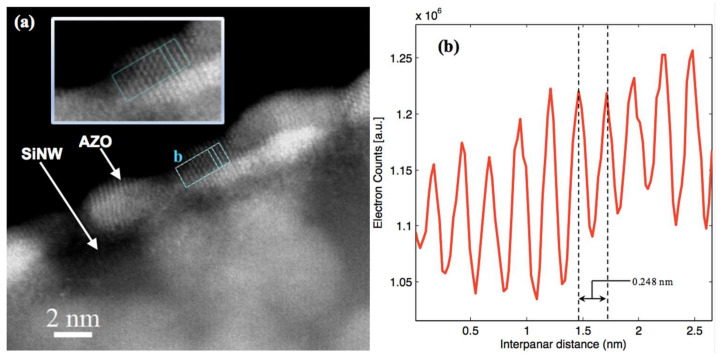
(**a**) High-resolution STEM image for 2 nm thick AZO layer deposited over one Si-NW. (**b**) Signal intensity profile of the AZO signal extracted from the area selected by the light blue box on a grain. The inset is a magnification of the analysed AZO crystalline grain.

The lattice parameters and the degree of crystallinity of the deposited AZO are important information because they determine its optical and electrical characteristics. Figure 3a shows the high-resolution STEM image of an Si-NW deposited in the same conditions used for the previous sample, i.e., with 2 nm of AZO equivalent thickness (as in Figure 2). The lattice planes of the clusters were clearly distinguishable (see the inset, which is a magnification of one of the crystalline AZO grains), demonstrating their crystalline nature. The nanocrystals were oriented in different directions from each other. The signal intensity profile shown in Figure 3b was extracted from the light blue box shown in (a). The measured interplanar distance for the specific crystallographic direction was 2.48 Å, which is compatible with the wurtzite crystal structure, with the (100) planes having an interplanar distance equal to 2.488 Å [36,37].

**Figure 4:**
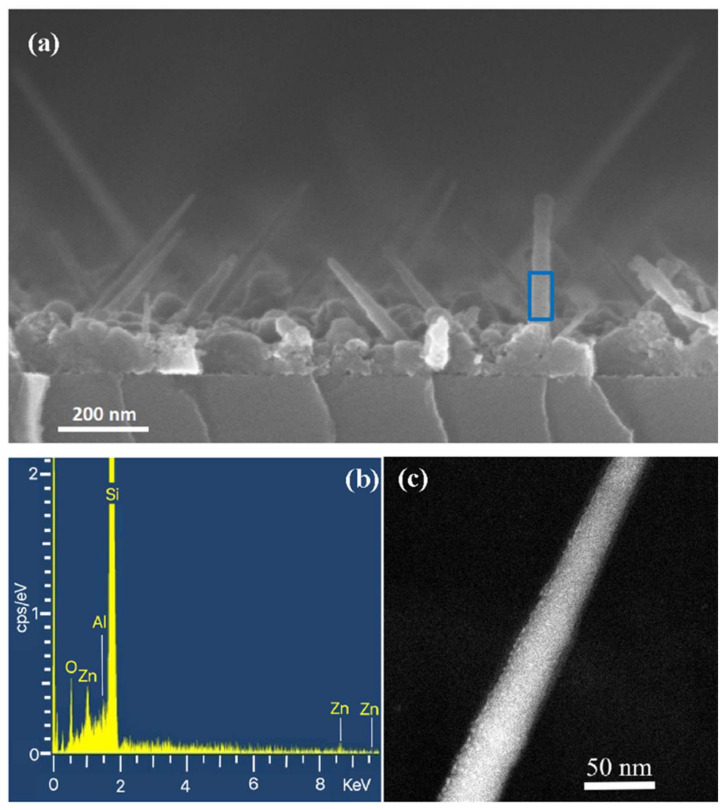
(**a**) Cross-SEM analysis of 20 nm thick AZO layer deposited on Si-NW. (**b**) EDX spectrum extracted from the NWs area referred to in letter a. (**c**) STEM micrograph of a single Si-NW coated with the equivalent 20 nm thickness of AZO.

The thickness of sputtered AZO was then increased to study the evolution of the system morphology in the subsequent deposition steps. Figure 4 shows the results for the sample on which the 20 nm thick AZO layer was deposited. The cross-SEM micrograph in Figure 4a shows that the SiNWs maintained their oblique orientation with respect to the substrate when the thickness of material deposited increased up to 20 nm. The polycrystalline Si layer at the base of the SiNWs was covered by a very thin AZO film, as demonstrated by local EDX analysis, not shown here. The blue rectangle indicates the area from which the EDX spectrum shown in Figure 4b was extracted. i.e., from one nanowire. It can be seen that the dominant species was Si, as expected. The presence of Zn, O and Al signals confirmed that the deposited shell was composed of AZO. The coverage, in any case, was uniform and conformal for the entire length of the wires and independent of the SiNWs tilt.

Figure 4c shows the STEM micrograph taken on the same sample, showing that the layer of AZO coated its entire surface. It allowed us to state that the clusters formed in the first deposition steps evolved into a continuous film onto the nanowires surface, probably by following a mechanism growth similar to “Island growth”, also known as “Volmer–Weber” [15], where the small clusters, nucleated separately on the Si-NW surface, grow as three-dimensional islands, and eventually coalesce to form a continuous film.

**Figure 5:**
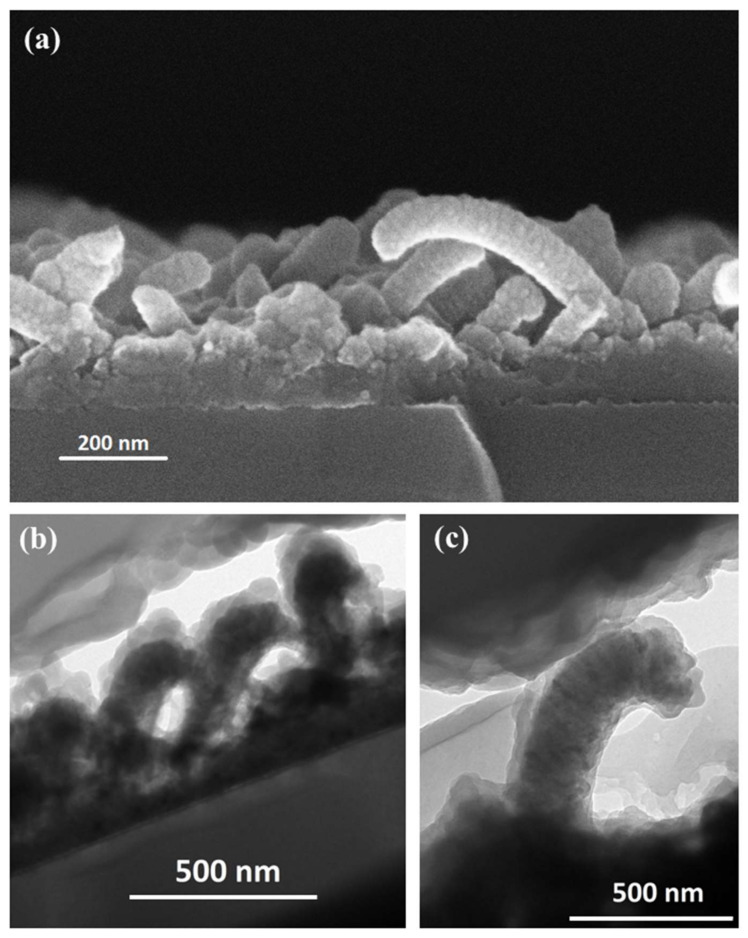
(**a**) SEM and (**b**,**c**) TEM microscopies in cross view of 100 nm thick AZO showing the conformal deposition of the oxide layer, the curved morphology of the nanostructure after the deposition and the inner silicon core with the AZO shell (**b**,**c**).

Figure 5 shows the SEM micrographs (a) and TEM (b,c) in cross view of AZO with a nominal thickness of 100 nm. It could be immediately observed that there were no empty spaces in the AZO shell. The most striking difference from the previous cases was that the core-shell SiNWs-AZO appeared curved. To understand SiNWs-AZO bending observed in Figure 5, we explored several possible explanations. The effect of the weight force exerted by the AZO mass deposited (of the order of 10^−14^ g per wire, making the hypothesis of a cylindrical wire 30 nm large, 450 nm long and a total diameter SiNWs + AZO equal to 130 nm) can be excluded, since the wires’ Young’s modulus reported in the literature for silicon nanowires of similar size is of the order of one hundred GPa [38]. Previous work shows the bending of SiNWs after the implantation of Ga ions accelerated at energies of the order of tens of KeV [39]. In that case, the bending was attributed to structural changes in crystallinity following implantation and not to the momentum transferred from the Ga ions to the wires. In our case the AZO deposition was done at 50 W, with an acceleration voltage of about 0.1 kV, so that a bending of the SiNWs as a consequence of the AZO impact was not probable. The possible strain effect exerted by the thick AZO onto the much thinner Si-NW, causing the elongation/deformation of the Si crystalline cell, could also be excluded because the AZO shell was polycrystalline, as already demonstrated. A possible explanation could be the effect of the magnetic field on the piezoelectric AZO: its effect could be reset at the end of the deposition on the thin layers, but not on the thick ones, because those could ‘encage’ the SiNWs core inside a rigid shell.

This observed phenomenon could have interesting applications because this particular morphology can be used as a tool to control both the electrical conductivity as well the optical properties of the system. Indeed, the literature reports that the photo-response performance increases by bending ZnO nanowires [40,41].

It should be noted that, by increasing the nominal thickness from 20 to 100 nm, the two-dimensional AZO film deposited at the interface with the substrate, between the SiNWs, did not significantly increase its thickness. Decreasing a material dimension to the nanoscale, the surface/volume ratio, as well as the dangling bond density increased, resulting in a more favored adhesion of AZO on SiNWs [42,43]. These sites were at higher energy than they were in the ideal crystalline lattice. In this way, it could be explained why the AZO thickness was greater on the Si-NW surface than on the substrate. We also point out that, already in these deposition conditions, without further increasing the AZO thickness, the nanowire array was embedded in the AZO layer. The structure could then guarantee, in a device where the Si-NW array works as an emitter, the electrical contact over the nanostructured layer.

**Figure 6:**
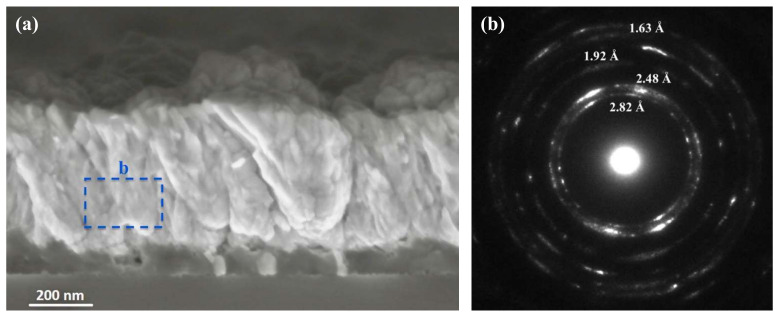
(**a**) SEM analysis in cross view of 500 nm AZO thick sample. (**b**) Diffraction pattern by electron beam carried out on to an area of the AZO layer as indicated by the dashed box in (**a**).

As the sputtering time further increased, the amount of material grew and the morphology of the system changed. Figure 6a depicts the cross-SEM of the 500 nm thick AZO sample. A planarized polycrystalline AZO matrix, with the embedded SiNWs, was produced. From these observations, it could be deduced that the sputtered material initially settled on the wires and on the Si at the interface, but that the production of the thick AZO layer did not start before all wires has been conformally and fully coated. The diffraction patterns shown in Figure 6b were carried out on a large area of the deposited bulk AZO matrix. Stretched spots inside the diffraction pattern were present, indicating that AZO was poly-crystalline and that the domains consisted of several smaller crystals with preferential crystallographic orientation. The interatomic distances measured from the diffraction pattern, 2.82 Å, 2.48 Å, 1.92 Å and 1.63 Å, corresponded to those of the ZnO in the wurtzite phase. In particular, the distance of 2.82 Å (<101> plane in wurtzite) did not appear among those of the zinc-blende; therefore, the possibility that the AZO was in this phase can be excluded. The crystallinity obtained was an encouraging result for good electrical conductivity, which is needed for any electronic applications. As the deposited AZO thickness increased, i.e., when the AZO clusters coalesced to form a compact shell, our preliminary electrical investigations—not reported here—showed that the electrical conductivity increased, although a deep investigation about electrical properties of the systems is still in progress.

## 4. Conclusions

In this work, we investigated the morphology of AZO on SiNWs, to understand its morphological and chemical evolution when deposited on substrates with complex shapes. Different nominal oxide thicknesses from 2 up to 500 nm were deposited. The 2 nm thick AZO turned out to be composed of nanoclusters distributed on the wire walls. Aluminum was detected even inside these initial small nanoclusters. The structure analysis revealed that the clusters were crystalline, with an interplanar distance that corresponded to that of the lattice in wurtzite phase. The 20 nm thick specimen showed a conformal two-dimensional AZO shell encapsulating the wires, probably produced according to the Volmer–Weber growth mechanism. By increasing the deposited oxide thickness, the AZO coating on the wires maintained the two-dimensionality without the appearance of empty spaces within it. With increasing the deposition thickness from 20 nm to 100 nm, we found that the thickness of the AZO film, deposited onto the Si substrate between the SiNWs, remained almost constant. This suggests a greater affinity of AZO for the Si-NW surfaces. A noteworthy aspect is that oxide-coated SiNWs, with nominal thickness of 100 nm, appeared hook-shaped after the deposition process. In the literature, it has been reported that the photo-response performance increased by bending ZnO nanowires; this aspect could be significant for their applications. The deposited AZO material exhibited a wurtzite crystalline structure, from the very early stages up to the bulk deposition. Bent composite structures, as well as the cluster decorated nanowires, can find applications in advanced plasmonic and optical systems.

## Data Availability

Data presented in this article are available upon request to the corresponding author.
